# Characterization of Municipal Solid Waste as Potential Fuel for Energy Needs

**DOI:** 10.3390/ma18092103

**Published:** 2025-05-03

**Authors:** Monika Uler-Zefikj, Katarzyna Godyń, Katarzyna Tokarczyk, Risto V. Filkoski

**Affiliations:** 1 Ss. Cyril and Methodius University in Skopje, Faculty of Mechanical Engineering, 1000 Skopje, Republic of North Macedonia; risto.filkoski@mf.edu.mk; 2 Strata Mechanics Research Institute, Polish Academy of Sciences, 30-059 Krakow, Poland; godyn@imgpan.pl (K.G.); katarzyna.tokarczyk@imgpan.pl (K.T.)

**Keywords:** calorific value, incineration, MSW, proximate analysis, solid waste, ultimate analysis

## Abstract

The continued expansion of cities in economic, population and geographical terms leads to significant environmental and infrastructural pressures, including the need for efficient municipal solid waste (MSW) management. This research focuses on the characterization of MSW generated in the city of Skopje and the investigation of its thermo-physical properties and energy utilization potential. The analyses cover physical and chemical properties, including density, moisture content, volatile matter, ash, and higher heating value, using adequate testing methods. The results indicate that MSW has a relatively high gross calorific value, surpassing typical MSW ranges comparable to those of solid fossil fuels. With approximately 79.42% volatile matter and a low ash content of 7.76%, the considered MSW demonstrates excellent combustibility. Chemical analysis reveals high carbon (53.12%) and hydrogen (7.69%) levels, supporting high energy value, while low nitrogen (0.84%) and sulfur (0.26%) levels ensure minimal NO_x_ and SO_x_ emissions. These characteristics position MSW as a suitable feedstock for energy production in incineration facilities. However, the heterogeneous composition of MSW presents challenges to process stability, necessitating prior waste preparation. The research concludes that harnessing waste energy potential could contribute to sustainability, reduce reliance on fossil fuels, and improve the environmental conditions in large urban areas.

## 1. Introduction

In a world that faces a continuous increase in local, regional, and global pollution and an urgent need for sustainable sources of energy, millions of tons of waste are generated every year, a significant part of which ends up in landfills.

Motivation for conducting this research stems from the challenges faced by the city of Skopje regarding waste management and air pollution [1]. The city continues to expand both economically and demographically, facing significant environmental and infrastructural pressures related to collection, treatment, and disposal of municipal solid waste (MSW) [2]. Waste management presents a major challenge, considering the continuously increasing amounts of waste generated, low public awareness of proper waste handling, and the incomplete practical implementation of the legislation for treatment of various waste types [3].

Understanding the composition and characteristics of MSW is essential for developing efficient waste management practices aligned with contemporary sustainability goals [4]. Characterization involves analyzing the types, quantities, and properties of the waste generated, which can contribute to an appropriate selection of treatment and processing methods. This information is vital not only for optimizing waste management but also for exploring its potential as a resource, especially in energy production [5]. Waste-derived materials, such as MSW incineration of bottom ash, have been shown to undergo structural and textural modifications over time, making them viable for various applications beyond disposal. Properly treated bottom ash can be repurposed in construction and environmental applications, reducing the environmental footprint of waste management [6]. Additionally, research has shown that the pore structures in incineration by-products, such as slags and ashes, share similarities with natural sedimentary rocks, making them potential candidates for reuse in construction materials [7].

The potential of MSW as an energy source has garnered global attention, with the application of various technologies such as incineration, anaerobic digestion, and gasification for converting waste into energy [8]. Also, according to studies, waste incineration can significantly reduce landfill pressures while recovering valuable energy from combustion [9]. Utilizing energy from waste can diversify the city’s energy portfolio by reducing dependence on traditional fossil fuels, minimizing landfill usage, and lowering greenhouse gas emissions [10]. The cement industry in Skopje has been integrating refuse-derived fuel (RDF) as an alternative to traditional fossil fuels. A study found that substituting RDF for coal and lignite in cement production leads to environmental benefits by reducing CO_2_ emissions [11]. For large urban areas, taking the city of Skopje as a case study, the waste-to-energy (WtE) approach can be transformative, addressing both waste disposal and energy needs simultaneously. A case study from Croatia highlights the potential of municipal solid waste for combined heat and power (CHP) generation, demonstrating that WtE systems can contribute significantly to both electricity and thermal energy production while enhancing overall energy sustainability [12].

However, a prerequisite for any WtE initiative is a comprehensive understanding of the characteristics of waste streams specific to the city. A study shows that seasonal variations affect the physico-chemical properties of MSW, revealing fluctuations in composition and emphasizing the need for seasonally adaptive waste management strategies to optimize material recovery and energy production [13]. Factors such as organic content, calorific value, combustible components, ash and moisture content, and the presence of volatile substances directly influence the choice of energy conversion methods and the efficiency of energy production [14].

This paper aims to provide a detailed characterization of MSW generated in the city of Skopje and examine the feasibility of utilizing this waste as fuel in waste incineration facilities for energy purposes. Although waste is often considered a non-usable material, its application for energy purposes transforms it into a renewable resource, contributing to the circular economy by naturally regenerating resources and reintegrating them into the process through renewable energy generation [15]. By identifying the primary components of waste, this paper seeks to offer strategic insights that could support sustainability in larger urban environments. The findings are expected to promote environmental management and encourage the adoption of integrated WtE systems that support both ecological and economic goals.

## 2. Materials and Methods

**Figure 1:**
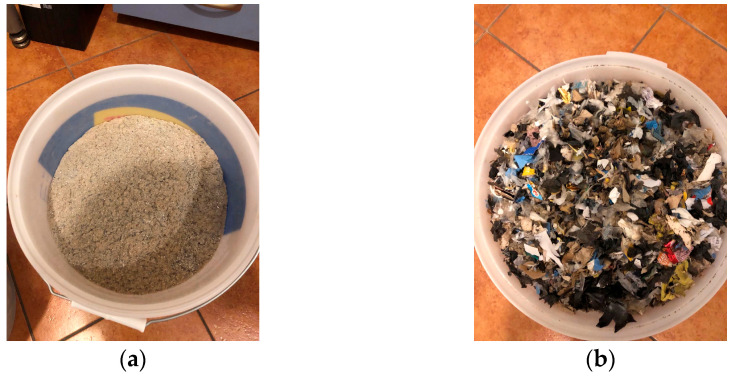
(**a**) Dry MSW sample MacM2; (**b**) dry MSW sample MacM30.

The methodological approach included various laboratory analyses of MSW from the city of Skopje to determine its potential use as fuel in the incineration process for generating electrical and thermal energy. The waste sampling was carried out according to the Standard CEN/TR 15310-1 Characterization of waste—Sampling of waste materials, Part 1: Guidance on selection and application of criteria for sampling under various conditions. The “composite sampling” strategy was applied, considering that it includes taking multiple subsamples combined to form a representative sample. The MSW originated from the one and only legal landfill in the country, the Drisla landfill, located in the south-eastern part of the city at a distance of 14 km from Skopje city center. The MSW sampled was packed in three polyethylene bags with a volume of 100 L. The approximate weight was 90 kg. The time period from the day of sampling till the days of the analyses was 5–10 days. Before analyzing the thermo-physical properties of the MSW, the samples were prepared in the laboratory of Ekospalarnia thermal waste treatment plant in Kraków, Poland, under controlled conditions. The preparation involved removing inert materials, shredding to reduce particle size, and drying to lower moisture content. After emptying the waste bags, a visual inspection was conducted to remove glass and metal, which are inert and could interfere with grinding. The first shredding stage reduced waste to fragments under 30 mm using a Radwag scale, conveyor belt, and Testchem shredder. Waste was weighed, manually transferred, and fed into the shredder via the conveyor belt. To further reduce particle size, the waste was first dried in a Pol-Eko Aparatura muffle furnace, which used radiative heating without direct flame contact. After drying, a Testchem LMN-240 knife mill was used to reduce the waste to a fraction of up to 10 mm. It was manually fed into a conical hopper, where it fell into the mill for further shredding. The final stage used a Testchem LMN-100 shredder to achieve a minimum particle size of 2 mm. These steps were essential, as certain equipment for analysis required a maximum particle size of 30 mm (designated as MacM30), while other equipment required a finer size of up to 2 mm (designated as MacM2). Both MSW fractions are shown in Figure 1. The analyses of the thermo-physical properties of dry MSW involved determining density, water permeability, volatile matter content, moisture content, the presence of mineral matter, pH value, carbon, hydrogen, nitrogen, and sulfur content, as well as the higher heating value (HHV). Section 2 presents the procedures and equations for conducting the analyses, Section 3 presents the key findings of the study, Section 4 provides an interpretation of the results and explains their significance, and Section 5 summarizes the key results.

The density of the MSW sample was determined using a gas pycnometer, specifically the AccuPyc II 1340 by Micromeritics located in Norcross, GA, USA. This instrument uses helium displacement to measure the volume of solid samples of irregular or regular shapes, calculating density by measuring the change in helium pressure within calibrated chambers. The atmospheric conditions were monitored during the analysis, and the ambient temperature in the laboratory was 20 °C. A precision test was conducted using a 51.085527 cm^3^ standardized sphere in a 100 cm^3^ steel chamber, ensuring accuracy through nine consecutive measurements within an acceptable error range. The chamber volume was chosen based on the waste sample size, ensuring it nearly filled the chamber to optimize measurement precision. Once validated, the sphere was removed, and the chamber was cleaned. The density analysis of the dry MSW was performed on two fractions: 2 mm and 30 mm. Samples were purged with helium and measured ten times, with density calculated as mass divided by volume as per Equation (1) [16].

(1) $$D_{sample}=\frac{M_{sample}}{V_{sample}}$$

*D_sample_*—Sample density [g/cm^3^];

*M_sample_*—Sample mass (inserted manually; sample is measured by balance) [g];

*V_sample_*—Sample volume, according to Equation (2) [cm^3^].

(2) $$V_{sample}=V_{cel}-V_{exp}\cdot \left(\frac{p_{1}}{p_{2}}-1\right)$$

*V_cel_*—Sample chamber volume (in this case 100 cm^3^);

*V_exp_*—Expansion chamber volume [cm^3^];

*p*_1_—Gauge pressure after fill [psig];

*p*_2_—Gauge pressure after expansion [psig].

**Figure 2:**
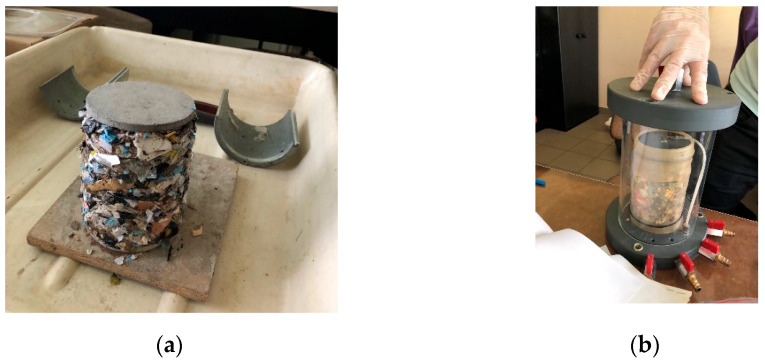
(**a**) Compacted waste outside cylinder; (**b**) waste placed in pressure chamber.

**Figure 3:**
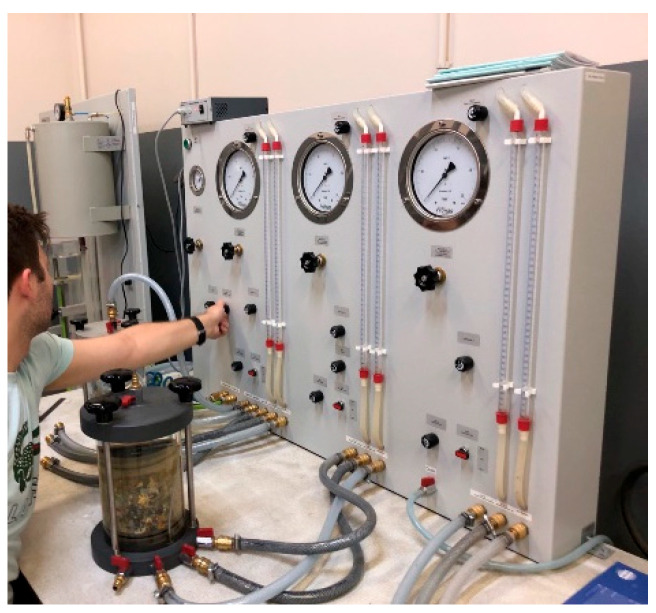
Pressure chamber connected to Frowag type 2.924.

The water permeability of the MSW was determined on a dry MSW fraction with a particle size of up to 30 mm, due to the suitability of the particle size for analysis. For a fraction of up to 2 mm, it was impossible to achieve a compact structure for the pressurized chamber due to the fine, powdery nature of the waste. The shredded solid MacM30 waste underwent compaction using a Proctor apparatus manufactured by MULTISERW from Brzeźnica in Poland. It was composed of a cylindrical mold volume of 7850 mm^3^ and a hammer weighing 2.50 kg, mounted on a shaft. The process began with assembling the metal cylinder and filling it with pre-mixed waste and water added in layers compacted with 25 blows of the hammer until the cylinder was filled to its full height [17]. The compacted waste form is shown in Figure 2a. The formed mold was then inserted into a rubber casing, placed in a pressure chamber (shown in Figure 2b), and connected to a Frowag type 2.924 apparatus (presented in Figure 3). The manufacturer of this apparatus is Fröwag GmbH from Obersulm-Eschenau in Germany. The apparatus uses a triaxial pressure system to test the sample’s water permeability at a working pressure of 2.5 bars and consists of a water supply tank, a pressure chamber for the sample, water sources, two burettes, and manometers for adjusting hydrostatic pressure and regulating fluid pressure at the sample’s inlet and outlet. The water permeability coefficient was taken as the arithmetic mean of the results obtained from two flow directions through the sample. During the experiment, the water temperature was controlled, and the difference in water levels in the burettes was measured. The applied sample pressures were 0.2 bar, 0.3 bar, 0.5 bar, 0.8 bar, and 1.0 bar, corresponding to depths ranging from 2.0387 m to 10.1937 m. The chamber pressure was selected to prevent significant penetration through the sample membrane or water extraction from the sample’s pores during the permeability test. Attention was given to maintaining the sample’s water content during the preparation process. The calculation of the water permeability coefficient of the dry MSW was carried out using Equation (3) [18].

(3) $$k=\frac{a_{in}\cdot a_{out}}{\left(a_{in}+a_{out}\right)}\cdot \frac{l}{A\cdot \Delta t}\cdot \ln\left(\frac{\Delta h_{t1}}{\Delta h_{t2}}\right)$$

*A* = 78.54 cm^2^—Cross-sectional area of the specimen in the flow direction;

*a_in_*—Cross-sectional area of the inlet pipe [cm^2^];

*a_out_*—Cross-sectional area of the outlet pipe [cm^2^];

Δ*h_t_*_1_—Head of water above the outlet elevation at time *t*_1_ [cm];

Δ*h_t_*_2_—Head of water above the outlet elevation at time *t*_2_ [cm];

*l* = 10 cm—Distance between measuring points 1 and 2 in the flow direction;

Δ*t*—Increment of time between two readings [s].

The moisture content, volatile matter, and ash were analyzed for only one fraction of the sample, dry MSW with particle sizes of up to 2 mm (MacM2). The reason for this was that the containers used for the analysis were small, and the smaller fraction provided a more homogeneous sample over a smaller surface area.

**Figure 4:**
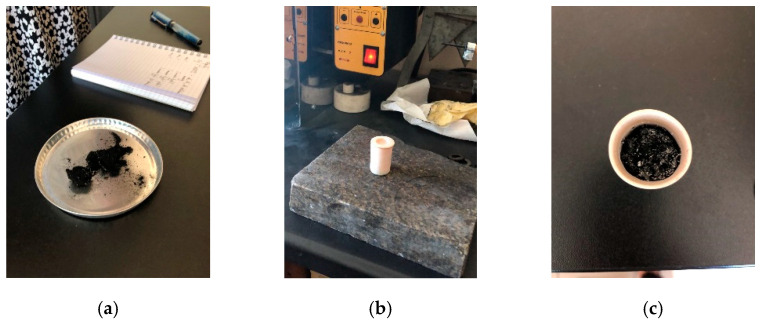
(**a**) Waste material after drying; (**b**) heated container containing waste after removal from the furnace; (**c**) waste in the container after removal from the furnace.

The moisture content in the sample was determined using a laboratory drying machine (dryer) from PPHU Zalmed located in Warsaw, Poland. The type of dryer was SML 32/250 with a maximum heating temperature of 250 °C, a power of 1000 W, and a height of 60 cm. The process began by setting the dryer temperature to 105 °C. A portion of the shredded and dried MSW sample of up to 2 mm was placed in a measuring dish and inserted into the dryer for 24 h. The condition of the waste after drying is shown in Figure 4a. The moisture content in the analyzed sample was calculated as the ratio of the difference in waste mass before and after drying to the mass of the waste introduced for the analysis, as described by Equation (4) [19].

(4) $$W=\frac{M_{2}-M_{3}}{M_{2}-M_{1}}\cdot 100$$

*M*_1_—Mass of the empty tray [g];

*M*_2_—Mass of the tray plus sample before drying [g];

*M*_3_—Mass of the tray plus sample after drying [g].

The presence of volatile matter was examined using a NT 1313 furnace from the Neoterm company from Varese in Italy. The furnace has a heating chamber volume of 4.5 dm^3^ and a maximum heating temperature of 1100 °C. The procedure began by adding a shredded and dried waste fraction of up to 2 mm into a container with a lid, which was placed in the furnace preheated to 900 °C and kept for 7 min. After stabilizing at room temperature, the volatile substance content was determined using Equation (5) as the ratio of the waste mass before and after heating to the mass of the introduced waste with the previously determined moisture content subtracted from it [20]. The red-hot state of the container upon removal from the furnace can be seen in Figure 4b, while the incinerated waste is shown in Figure 4c.

(5) $$VM=\frac{100\left(m_{2}-m_{3}\right)}{m_{2}-m_{1}}-W$$

*m*_1_—Mass of the empty crucible and lid [g];

*m*_2_—Mass of the crucible and lid and test portion before heating [g];

*m*_3_—Mass of the crucible and lid and contents after heating [g];

*W*—Moisture in the sample as analyzed [percentage mass fraction].

To determine the ash content or organic matter in the MSW sample, the same Neoterm NT 1313 furnace was used, but with a different procedure. The dry MacM2 waste sample was heated at 800 °C for 1 h, then stabilized at room temperature and weighed. This process was repeated until a constant mass was reached. The ash content was calculated based on the mass difference between the initial drying hour and the n-th hour from the mass difference between the initial drying hour and the empty container from the whole. This statement is represented by Equation (6) [21].

(6) $$A=\left(1-\frac{m_{2}-m_{5}}{m_{2}-m_{1}}\right)\cdot 100$$

*m*_1_—Mass of the empty crucible and lid [g];

*m*_2_—Mass of the crucible and lid and test portion before the 1st hour of heating [g];

*m*_5_—Mass of the crucible and lid and test portion after the 3rd hour of heating [g].

**Figure 5:**
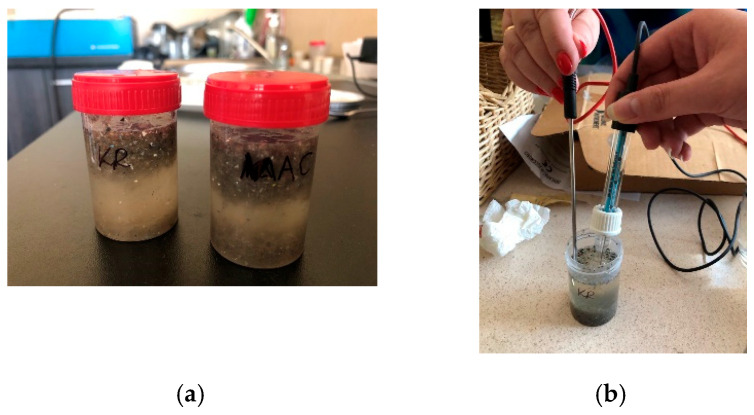
(**a**) Mixture of waste and water; (**b**) pH value measurement procedure.

The pH value was measured using a pH meter from Elmetron from Zabrze in Poland. The type of the pH meter was CP-411 equipped with an ERH-11S electrode, with a measuring range of 0–14 pH and a temperature range of 0–60 °C. The sample preparation for analysis was carried out by mixing shredded and dried MacM2 waste with water in a plastic container and leaving it to stabilize for 24 h, allowing for particle separation (as shown in Figure 5a). The electrode was immersed in the solution for 1 min, after which a stable pH value was obtained [22]. The pH measurement process of the waste is presented in Figure 5b.

The contents of carbon, hydrogen, nitrogen and sulfur, as well as calorific value, were analyzed using different analyzers from the company LECO Polska SP. Z O.O. located in Tychy in Poland.

The carbon, hydrogen, and nitrogen content was determined using a LECO analyzer, model Truspec CHN 628, with detection ranges for carbon from 0.002 to 100%, for hydrogen from 0.02 to 50%, and for nitrogen from 0.02 to 50%, with an analysis time of approximately 4 min. The process was carried out by introducing shredded and dried MacM2 waste into a combustion chamber, where it burned in the presence of pure oxygen at 950 °C. During combustion, the carbon in the sample was converted to carbon dioxide (CO_2_), hydrogen to water vapor (H_2_O), and nitrogen to nitrogen gas (N_2_) and nitrogen oxides (NO_x_). The quantities of carbon and hydrogen were determined based on the produced CO_2_ and H_2_O gases. From the measurements, the percentages of carbon, hydrogen, and nitrogen in the sample were calculated using stoichiometric relationships [23].

The HHV of the shredded and dried solid waste was determined using the LECO AC500 calorimetric bomb. The MacM2 sample was placed in a combustion container inside a sealed calorimetric bomb, filled with oxygen at ≈30 bar and submerged in bucket with a softened water whose temperature was recorded every 6 s. The analysis included a 3 min equilibrium phase for establishing thermal balance between the water and the bomb, followed by a 5 min controlled combustion. The heat released during combustion was calculated from the system’s thermal balance [24].

For laboratory testing of sulfur in the MacM2 waste sample, the LECO S832 analyzer was used, with a sulfur detection range of 0.008 to 30 mg and an analysis time of 60 to 120 s. The analysis began by introducing a small amount of waste into the furnace preheated to 1350 °C containing pure oxygen. Moisture and other impurities were removed from the sample, as it had been previously dried. Combustion released carbon in the form of CO_2_ gas and sulfur in the form of SO_2_ gas, with additional oxygen introduction to accelerate refractory material combustion [25].

## 3. Results

The results were obtained based on equations in compliance with the standards for conducting the specified analyses, as described in the previous section.

**Figure 6:**
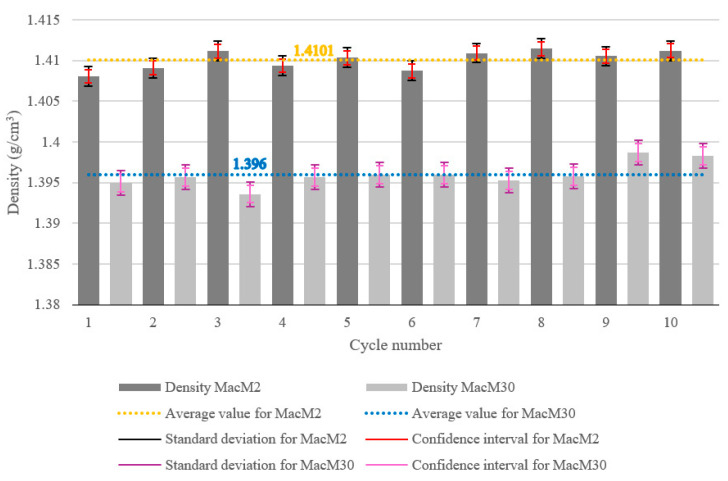
Density of the dry MSW analyzed for two fractions across 10 consecutive measurements.

The density measurements for MacM2 and MacM30 over ten cycles are presented in Figure 6. The density of MacM2 remained consistently higher than that of MacM30 across all cycles. The average density for MacM2 was 1.4101 g/cm^3^ (dotted orange line), while MacM30 had a lower average density of 1.396 g/cm^3^ (dotted blue line).

The standard deviations (SDs) of the density measurements of the dry MSW are marked with a black double-compound-type line for the MacM2 sample and a purple-colored line in the same style for MacM30. The SD of MacM2 was ±0.00119 g/cm^3^ and of MacM30 was ±0.00149 g/cm^3^, meaning that smaller particles exhibited higher consistency in the data. This can be explained by the greater uniformity, more efficient packing, and reduced random variability in smaller waste. The confidence intervals (CIs) of both waste samples are also shown in Figure 6, with red bars for MacM2 (±0.0008 g/cm^3^) and pink bars for MacM30 (±0.0011 g/cm^3^). This is consistent with the SD results, where smaller waste particles also had a lower CI for similar reasons. Additionally, it can be noted that the SD intervals were greater than the CI intervals, which means that the individual data points were widely spread around the mean, leading to high dataset variability, while the narrower CI interval means the average value was estimated with high precision, likely due to the considerable number of samples (cycle number) reducing uncertainty in the mean.

**Figure 7:**
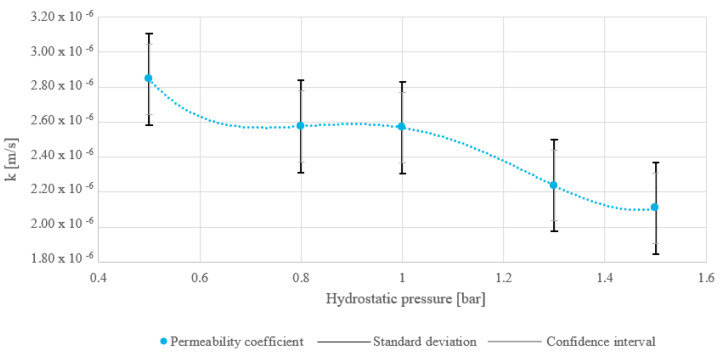
Water permeability coefficient for the dry MSW analyzed for one fraction of MacM30.

The water permeability coefficient results are provided in Figure 7 and ranged in interval from 2.11 × 10^−6^ m/s to 2.84 × 10^−6^ m/s. The permeability coefficient decreased as hydrostatic pressure increased, indicating that higher pressure reduced the material’s permeability. This trend suggests compression of the material’s pore structure, reducing the ability of fluids to pass through. Also in Figure 7, the SD is marked with a black double-compound-type line and has a value of ±2.627 × 10^−7^ m/s, and the CI range is labeled with gray bars and amounts to ±2.020 × 10^−7^ m/s. Since only one measurement was available per pressure point, the SD was calculated from the average of all pressure measurements rather than individually for each point. While this approach assumes consistent variability across all pressures, it provides a reasonable estimate in the absence of multiple replicates per condition. However, future studies would consider obtaining multiple measurements per pressure to evaluate pressure-dependent variability more accurately.

The moisture content, volatile matter, ash, and pH were analyzed only for waste sample MacM2, and the results are provided in Table 1. The moisture content of the analyzed MSW sample was relatively low, measured to be 2.76%, indicating that the process of drying the sample was performed prior to the analysis. The volatile matter content was 79.42%, suggesting a high proportion of combustible organic components. The mineral matter or ash content was 7.76%, representing the content of inorganic, non-combustible fraction of the waste. Additionally, the pH value of the waste sample was 7.51, indicating a neutral to slightly alkaline nature. This suggests a moderate presence of mineral compounds (e.g., carbonates), which may affect thermal treatment processes. However, while some minerals contribute to slagging, carbonates typically decompose into high-melting oxides that may not directly promote slag formation.

**Figure 8:**
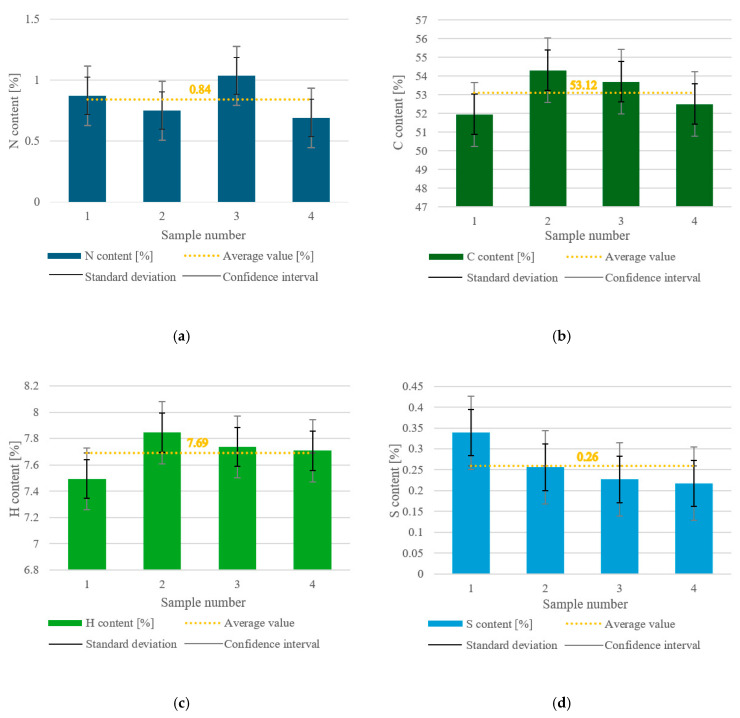
(**a**) Nitrogen content in the dry MSW; (**b**) carbon content in the dry MSW; (**c**) hydrogen content in the dry MSW; (**d**) sulfur content in the dry MSW.

The values for nitrogen, carbon, hydrogen, and sulfur content were analyzed for only one fraction of the sample, with particle sizes of up to 2 mm (MacM2), and are shown in Figure 8a–d, respectively. In (a) to (d) in the figure, the average value is shown with an orange dotted line, the SD is marked with a black double-compound-type line, and the CI is labeled with grey bars.

The nitrogen content varied from approximately 0.7% to 1.04% across the four samples, and the average content was 0.84%. The SD had a value of ±0.15%, whereas the CI range was within ±0.24%. The SD was about 18% of the average value, which suggests moderate variability in the nitrogen content across the samples. The CI was about 29% of the average, which tended to be wide and led to some uncertainty in the estimate (likely due to the limited number of measurements).

The carbon content ranged from 51.47% to 54.31%, and the average value was 53.12%. The SD interval was ±1.08%, and the CI range was ±1.72%. The SD was about 2% of the average, which suggests a low amount, indicating that the results were more tightly clustered around the average value. The CI was around 3% of the average, showing low to moderate uncertainty.

The content of hydrogen in the dry MSW sample was in the interval of 7.49% to 7.84%, with an average value of 7.49%. The SD was ±0.15%, indicating low variability in the hydrogen content among the samples, and the CI was ±0.23% showing a relatively narrow range, meaning high reliability of the data.

The sulfur content varied in the range of 0.217% to 0.339%, with a mean value of 0.26%. The SD values were within the interval of ±0.055%, and the CI range was ±0.088%. The SD was 21.15% of the mean, showing moderate variability in sulfur content across the samples. The CI was 33.85% of the average, which is relatively wide, meaning there was uncertainty in estimating the true population mean. One reason for this occurrence is that when dealing with very small numerical values, even minor variations can result in a relatively high percentage of SD and CI compared to the mean.

The average values for all components (N, C, H, S) fell within the SD range. This consistency suggests that the variability in the data was not extreme, and the average was a reasonable representation of the dataset. However, only C was within the CI range, meaning that the estimate for C was statistically more precise and reliable compared to the other elements.

**Figure 9:**
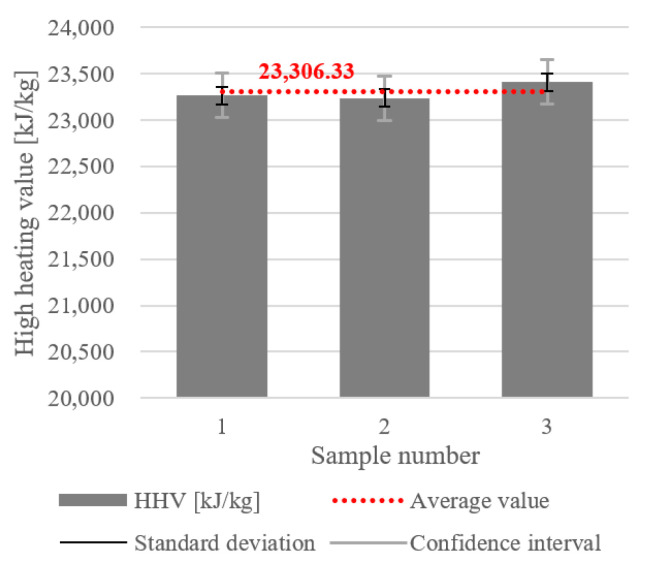
Higher heating value (or gross calorific value) of the dry MSW.

The results for the gross or higher calorific value are presented in Figure 9, and were also obtained for the MacM2 sample. The HHV values for the three samples were from 23,236 to 23,414 kJ/kg. The average HHV was 23,306.33 kJ/kg, indicated by the red dotted line in the Figure. The SD of ±94.694 kJ/kg was relatively small, indicating that the HHV values did not vary significantly between samples. However, the CI of ±235.24 kJ/kg was noticeably larger, suggesting some uncertainty in the estimate of the true mean HHV. The larger CI might have been influenced by a smaller number of measurements, minor heterogeneities in material composition, or measurement uncertainties or variations in experimental conditions.

The ultimate analysis of the dry MSW provided the quantitative amounts of its components: carbon (C), hydrogen (H), oxygen (O), nitrogen (N), combustible sulfur (S), mineral matter (ash) (A), and moisture (W). The ultimate analysis of the dry MSW is given in Table 2, and the proximate analysis of the MSW is given in Table 3.

The analyzed material exhibited high carbon (53.12%) and hydrogen (7.69%) content, indicating strong energy potential for thermal conversion, while the moderate oxygen content (27.57%) may have slightly lowered its calorific value. The low nitrogen (0.84%) and sulfur (0.26%) levels suggest low NO_x_ and SO_2_ emissions during combustion. Additionally, the ash content (7.76%) was low to moderate, reducing slagging concerns, and the low moisture content (2.76%) enhanced combustion efficiency. Overall, the material demonstrated good suitability for energy recovery, with low environmental impact (according to the parameters tested).

## 4. Discussion

From all the conducted analyses of the dry MSW, the following findings were obtained. The average density of the MSW with a smaller particles size (MacM2) was 1.4101 g/cm^3^, while the one with larger particles (MacM30) had a lower average density of 1.396 g/cm^3^. The reason for this might be packing efficiency and void space reduction. Smaller particles can fill gaps between other particles more efficiently, leading to higher packing density. Additionally, a finer fraction has more surface area per unit volume, allowing for better adhesion or cohesion, which can contribute to increased density, whereas larger particles create more voids and irregular spaces and are loosely arranged, reducing overall density.

The average density of both dry MSW samples (MacM30 and MacM2) was approximately 1400 kg/m^3^, significantly above the typical range for untreated waste, as outlined in the papers referenced in the following text. In high-income countries, MSW density typically ranges from 100 to 150 kg/m^3^; in middle-income countries, this density increases to between 175 and 330 kg/m^3^ [26,27]; while in low-income nations, it is estimated to be between 300 and 600 kg/m^3^ [28,29]. According to data from the State Statistical Office of North Macedonia, the composition of MSW generated is composed mainly of organic waste, with 49%; followed by plastic waste, with 19%; paper, with 15%; glass, with around 5%; metal and textiles, with less than 3%; and rubber, with less than 2% [30]. The high density value can be attributed to the large amount of organic waste present in the total MSW (almost half of its composition), considering that landfilled food waste has a density of 1300 to 1900 kg/m^3^, as reported in Ref. [31]. Waste density can influence its combustible characteristics indirectly. High density increases the energy output, as it contains more combustible components per unit volume. It is more compact, potentially improving consistency in fuel supply to combustion systems. However, denser waste can restrict airflow and retain moisture, requiring additional preprocessing before combustion.

The average permeability coefficient of dry MSW was about 2.47 × 10^−6^ m/s, indicating low to moderately low water permeability, which makes the material waterproof and minimizes the leakage of pollutants into surrounding soil and groundwater. However, low permeability increases moisture retention, reducing the calorific value, as reported in Ref. [32]. This necessitates prior waste preparation, such as drying, before feeding it into the furnace. Also, it can be noted that the water permeability coefficient of the material decreased as hydrostatic pressure increased (see Figure 9). This suggests that several phenomena might have occurred, as confirmed in Ref. [33], such as pore structure compression shrinking the water pathways, reduced pore connectivity blocking the channels, or structural or chemical changes induced in the material, altering its permeability properties.

The examined dry MSW had a higher calorific value of 23,300 kJ/kg due to its significant carbon and hydrogen content and low moisture content. This value exceeds the typical calorific range for MSW (7,000–15,000 kJ/kg) [34,35] and is comparable to the calorific value of high-quality solid fossil fuels [36]. One drawback of MSW compared to other fossil fuels is its inconsistent composition and heterogeneous structure, which can significantly affect its calorific value.

The average measured values for nitrogen, carbon, hydrogen, and sulfur in dry MSW were 0.84%, 53.12%, 7.69%, and 0.26%, and the SDs for each of these parameters was ±0.153%, ±1.082%, ±0.147%, and ±0.055%, respectively. Using the SD, the coefficient of variation was calculated for every parameter, amounting to ±18.2 for nitrogen, ±2.0 for carbon, ±1.9 for hydrogen, and ±21.3 for sulfur. These values provide insight into the relative variability of the results of each sample for different parameters and help assess measurement reliability [37]. Carbon and hydrogen had low variability, meaning that the measurements were precise and repeatable, and the MSW composition in terms of these parameters was highly consistent, whereas nitrogen and sulfur had moderate to high variability and showed some fluctuations between samples. The explanation regarding these differences in variability of different parameters is related to the concentration of each component in the sample, the heterogeneity of the sample, and measurement sensitivity. Carbon and hydrogen made up a large proportion of the sample composition, leading to more stable measurements. Also, they are structural components of organic matter in the MSW, meaning they were consistently present. External factors had a lower influence on their levels in the MSW. Sulfur and nitrogen were present in much smaller amounts than carbon and hydrogen, and even small changes in their content created large relative variations. The heterogeneity of the sample impacted their content. If the sample had contained more plastics, the sulfur content would have been greater, whereas a higher proportion of food waste would have resulted in a higher nitrogen content, as stated in Ref. [38]. Also, the instruments used to detect smaller concentrations tended to have slightly higher error margins, increasing variability. The CIs for each parameter in the dry MSW were ±0.2426% for nitrogen, ±1.7210% for carbon, ±0.2347% for hydrogen, and ±0.0881% for sulfur. The interpretation would be that nitrogen, hydrogen, and sulfur had low uncertainty, suggesting more precise and reliable measurements, whereas carbon had the largest CI interval, meaning its measurements were less precise and fluctuated more. This might have been due to natural fluctuations in organic matter in the MSW.

The following paragraph highlights the environmental impact by addressing the potential exhaust emissions generated from the components present in the dry MSW during combustion. Low nitrogen content reduces the potential for forming nitrogen oxides (NO_x_), which, according to Ref. [39], results in less energy loss from these pollutants and leads to more stable and efficient combustion. The hydrogen content in the dry MSW was slightly higher than the typical value for MSW (4–6%), making it favorable for combustion, as it contributes to heat release during oxidation, as observed in Ref. [40]. As stated in Ref. [41], low sulfur content minimizes the production of sulfur oxide (SO_x_) emissions during combustion. Considering that this MSW had a sulfur content of 0.26%, this minimizes the risk of acid gas formation and corrosion in boilers and chimneys. It also lowers the need for desulfurization processes, thereby reducing operational costs. One of the most critical concerns in MSW incineration is the formation of highly toxic dioxins if chlorinated compounds are present in the MSW and if incineration temperature control is improper. Taking into account the composition of the MSW provided by the State Statistical Office of North Macedonia [30], as well as the ultimate and proximate analysis, the following assessment can be made. In the MSW sampled, the content of plastic waste was 19%, which is considered relatively high. Plastic waste increases chlorine availability, which reacts with organic matter during combustion to form dioxins. Paper waste and organic waste contribute to the carbon content in MSW. Considering that this MacM sample contained notable plastic and biowaste, with 15% and 49%, respectively, the carbon component is significant and serves as precursor for dioxin synthesis, as stated in Ref. [42]. Metals like iron and aluminum, present in this MSW in an amount of 2.50%, can act as catalysts for dioxin formation in fly ash [43,44]. However, there are factors related to this examined MSW composition that do not trigger the formation of dioxins. In Ref. [45], it is found that increased moisture content requires more energy for combustion, potentially leading to incomplete burning and increased dioxin formation. Considering that the MacM had low moisture content (2.76%), this occurrence was prevented. Also, the high presence of volatile matter in the MSW (79.42%) suggests complete combustion, reducing incomplete burn residues that promote dioxin synthesis [46].

The volatile matter content in the dry MSW was high, at 79.42%, making it suitable for rapid ignition, but it may lead to intense combustion. This requires controlled air supply to ensure complete oxidation and prevent the formation of unburned compounds. The low ash content of 7.76% indicates a smaller presence of inert materials, which reduces equipment fouling and clogging, leading to better heat transfer, as shown in Ref. [47], and lower maintenance costs. Additionally, the low ash content in the MSW resulted in a smaller quantity of ash as a byproduct, with potential for reuse in construction or other applications (if inert and non-toxic).

## 5. Conclusions

This study highlights the significant potential of MSW generated in large urban areas as a sustainable and efficient energy source for WtE systems. The key findings demonstrate that previously treated MSW exhibits favorable physical, chemical, and thermal properties, collectively positioning it as a viable alternative to traditional fossil fuels for energy applications, while maintaining a reduced environmental impact.

The density of dry MSW, at 1,400 kg/m^3^, is significantly higher than the average density of untreated waste, enhancing its energy output per unit volume. The low to moderately low permeability of 2.47 × 10^−6^ m/s minimizes the leachate leakage from landfills, thereby minimizing environmental risks. The calorific value of 23,300 kJ/kg is comparable to that of solid fossil fuels, and the favorable chemical composition—high in carbon and hydrogen, and low in nitrogen and sulfur—ensures efficient combustion of MSW, with minimal emissions of harmful pollutants such as NO_x_ and SO_x_. The high volatile matter content of 77% supports rapid ignition, while the low ash content of 9% minimizes residual production, reducing maintenance demands. These characteristics underscore its suitability for heat energy production, with minimal operational and environmental drawbacks.

In conclusion, the analyzed dry MSW is a feasible raw material for thermal energy facilities operating on waste incineration principles for generating electricity and heat energy, offering a sustainable pathway for energy recovery and waste management. However, to enhance its efficiency, further development of selection and pretreatment processes and optimization of combustion systems is recommended. Future research should explore the long-term environmental impacts, system integration, and process scalability to further validate the critical role of MSW in sustainable energy strategies.

## Figures and Tables

**Table 1:** Moisture, volatile matter, ash content, and pH values for the dry MSW.

Parameter	Mark	Result
Moisture content	*W*	2.76%
Volatile matter	*VM*	79.42%
Mineral matter (ash)	*A*	7.76%
pH value	*pH*	7.51

**Table 2:** Ultimate analysis of the dry MSW (in % mass).

N	C	H	S	O	A	W
0.84	53.12	7.69	0.26	27.57	7.76	2.76

**Table 3:** Technical (proximate) analysis of the dry MSW (in % mass).

A	C_fix_	VM	W
7.76	10.06	79.42	2.76

## Data Availability

The original contributions presented in this study are included in the article. Further inquiries can be directed to the corresponding author.

## Abbreviations

The following abbreviations are used in this manuscript:

MSW	Municipal solid waste
WtE	Waste to energy
HHV	Higher heating value (gross calorific value)
VM	Volatile matter
SD	Standard deviation
CI	Confidence interval
